# Selected Advances in the Anti-arrhythmic Management of Atrial Fibrillation: 2023

**DOI:** 10.19102/icrm.2024.15014

**Published:** 2024-01-15

**Authors:** James A. Reiffel

**Affiliations:** 1 Columbia University, New York, NY, USA

**Keywords:** Anti-arrhythmic drugs, anticoagulation, atrial fibrillation, catheter ablation, clinical trials, hybrid therapy, subclinical

*“The truth will set you free, but, first, it will piss you off.”* ^1^

As the Pharmacologic Insights Section Editor of the *Journal of Innovations in Cardiac Rhythm Management* (*JICRM*), I have the opportunity each year to write a year-end commentary for this section if one seems appropriate. For 2023, I have elected to chiefly comment upon the following: (1) the issue of oral anticoagulation (OAC) for patients with subclinical atrial fibrillation (SCAF) and (2) the delusive comparison of anti-arrhythmic drugs (AADs) and catheter ablation (CA) for the rhythm management of atrial fibrillation (AF) when, not infrequently, the real-world application is their use in combination with one another. For these, the quote I used as a preamble to this paper may be particularly applicable.

As this article is being written for the Pharmacologic Insights section of *JICRM*, I am not going to comment on updates to CA in 2023. However, I will point out that, common to both AAD and ablation trials, 2023 saw a growing appreciation that minimizing atrial myopathy by earlier rhythm control and by earlier management of contributing comorbidities can result in improved AF rhythm management, slowing of AF progression from paroxysmal (PAF) to persistent and permanent AF, and the reduction of adverse outcomes.^2–7^ Likewise, 2023 saw a growing appreciation that time to first recurrence, a very common primary efficacy outcome in clinical trials, is not particularly meaningful in the management of patients with AF. Rather, the reduction of overall events across time, the reduction in AF burden, and improvements in the quality of life are better metrics with respect to meaningful rhythm management, whether by AADs and/or ablation.^8,9^ Finally, 2023 noted continued growth in the appreciation that accurate measurement of AF burden and AF events (including asymptomatic episodes) requires continuous monitoring rather than intermittent electrocardiogram sampling.^8,9^ Derived from this is the understanding that the comparison of results across trials (cross-trial comparisons) may yield misleading interpretations if the monitoring methods used to assess outcomes differ among the trials—just as demographic differences, treatment differences, comorbidity differences, and disease pattern differences among the study groups can as well. Such an understanding may be particularly applicable to AAD versus CA trials for AF where AF type, the duration of AF history, underlying comorbidities, the degree of atrial myopathy, concomitant treatments, and monitoring methods have varied tremendously.

## Should patients with subclinical atrial fibrillation receive anticoagulation?

Many patients have AF that has not yet been clinically recognized, either through symptom evaluation or by electrocardiographic capture. The fact that such AF is present can be documented by wearable or insertable/implanted devices, such as smartwatches, pacemakers (PMs), implantable cardioverter-defibrillators, or inserted cardiac monitors. This kind of AF has been termed SCAF or less precisely defined as atrial high-rate episodes (AHREs) when device categorization is imprecise. For this discussion, SCAF should be distinguished from periods of asymptomatic AF in patients with clinically recognized AF. Importantly, SCAF has been prognostically associated with the same types of adverse outcomes linked to clinically recognized AF, eg, thromboembolism, death, and more, albeit with lower event rates.^10,11^ Similar to clinically evident AF, the risk for adverse outcomes with SCAF has been shown to relate to the type and severity of associated comorbidities (as represented by those that make up the CHA_2_DS_2_-VASc score^12^) as well as to the length of SCAF episodes/AF burden (though with some uncertainty as to what duration is of importance in which type of patient).^11^ What is not yet adequately defined is whether there is a threshold effect for either SCAF burden or a combination of SCAF burden and CHA_2_DS_2_-VASc score above which prophylactic anticoagulation should be utilized.^13^ The most recent major guidelines—those from the European Society of Cardiology released in 2020^12^—state that “very short episodes (≤10–20 s/day) are considered clinically irrelevant, as they are not significantly associated with longer episodes or an increased risk of stroke or systemic embolism. However, longer episodes of AHRE/subclinical AF (≥5–6 min) are [at least epidemiologically] associated with an increased risk of clinical AF ischemic stroke, major adverse cardiovascular (CV) events, and CV death. Overall, the absolute risk of stroke associated with AHRE/subclinical AF may be lower than with clinical AF.”^12^ Also, “whereas available evidence is insufficient to justify routine OAC use in patients with AHRE/subclinical AF, modifiable stroke risk factors should be identified and managed in each patient. The use of OAC may be considered in selected patients with longer durations of AHRE/subclinical AF (≥24 h) and an estimated high individual risk of stroke, accounting for the anticipated net clinical benefit and informed patient’s preferences.”^12^ This approach suggests a synergistic effect of AF burden and comorbidity severity rather than the effect of some arbitrary AF duration threshold, which is in keeping with their co-contributory action to promote atrial myopathy and its downstream effects.^13–15^ In a related fashion, SCAF lasting <24 h may be sufficient to cause adversities if both the number and severity of comorbidities are high.^11^ However, data-derived guidelines sufficient to determine any boundaries above which OAC should be initiated do not yet exist. To hopefully provide more definitive data with respect to this issue, several clinical trials, including Atrial Fibrillation Detected by Continuous Electrocardiogram Monitoring Using Implantable Loop Recorder to Prevent Stroke in High-Risk Individuals (LOOP),^16,17^ Non–Vitamin K Antagonist Oral Anticoagulants in Patients with Atrial High-rate Episodes (NOAH-AFNET 6),^18^ and Apixaban for the Reduction of Thrombo-embolism in Patients with Device-detected Sub-clinical Atrial Fibrillation (ARTESIA),^19^ were initiated. The highlights of these trials are presented later, but note: (1) in each trial, a significant number of patients had early termination due to withdrawal of consent, development of clinical AF necessitating non-blinded anticoagulation, death, or other reasons. However, the event rates shown for the primary analyses may not fully reflect these patient losses. (2) In NOAH-AFNET 6, AHREs were labeled as such rather than specified as SCAF; however, for the purpose of this discussion, I am considering them the same, as if the AHREs were SCAF.

LOOP^16,17^ enrolled older patients with at least one additional stroke risk factor (hypertension, diabetes, heart failure, prior thromboembolism) but no known history of AF. Patients were randomly assigned in a 1:3 ratio to receive an insertable loop recorder (ILR) versus usual care (control group). In the ILR group, OAC was recommended (but not mandated) if an AF episode of ≥6 min was detected. Thus, LOOP was not specifically a trial on OAC in SCAF. Of the 6004 patients enrolled, 1501 (25%) were randomized to the ILR group, of which 94.6% had a device implanted, and 4503 (75%) were randomized to the usual care group. The mean age was 74.7 years, 47.3% of participants were women, and 90.7% had hypertension. Twenty-eight percent had a prior history of stroke, systemic embolism, or transient ischemic attack. The number of patients with prior major bleeds was not provided. The median CHA_2_DS_2_-VASc score was 4 points. No patients were lost to follow-up, but 12.1% discontinued monitoring prematurely.

The primary outcome in LOOP was time to first stroke or systemic embolism as analyzed by time to first event. During a mean follow-up of 64.5 months with a median duration of ILR monitoring of 39 months, AF was diagnosed in 1027 participants, including 31.8% in the ILR group (34% of those with an ILR implanted) and 12.2% in the usual care group (hazard ratio [HR], 3.17; *P* < .0001). OAC was initiated in 29.7% of patients in the ILR group (93% of those with SCAF detected) and 13.1% of patients in the control group (HR, 2.72; *P* < .001). (Of the 591 control group patients in whom OAC was given, 550 had AF, while 41 received OAC for other reasons. Of the 550 with AF, 87% received OAC.) The primary outcome occurred in 4.5% of patients in the ILR group and 5.6% of patients in the control group (HR, 0.8; *P* = .11). Secondary outcomes were: (1) the combined endpoint of ischemic stroke, transient ischemic attack, or systemic arterial embolism; (2) the combined endpoint of stroke, systemic arterial embolism, or CV death; (3) CV death; and (4) all-cause death. Other outcomes included diagnosis of AF, the initiation of anticoagulation, and major bleeding. The secondary outcome of stroke, systemic embolism, and CV death occurred in 6.9% of patients in the ILR group versus 8.3% of patients in the control group (HR, 0.83; *P* = .10). Major bleeding occurred in 4.3% of patients in the ILR group and 3.5% of patients in the control group (HR, 1.26; *P* = .11). There were no differences in CV deaths or hemorrhagic stroke. Total mortality was approximately 11%. Thus, in the ILR group, there was approximately a threefold increase in AF detection (lasting ≥ 6 min) and OAC initiation. Yet, there was no significant decrease in stroke or systemic embolism.^16,17^

As there was no statistically significant difference in the primary outcome, these findings might imply that SCAF is not worth screening for and/or that OAC is not needed in SCAF-detected patients or that the trial was underpowered. However, it is possible that the greater number of patients taking OAC in the ILR group offsets the effects of a greater incidence of detected AF in the ILR group with regard to thromboembolism, which could suggest a benefit of OAC in some SCAF patients. Unfortunately, although the numerical event rates favored OAC, the size of the population limited demonstration of a benefit during follow-up, as the study was underpowered to detect a moderate treatment effect, such as a 20% difference. Thus, the LOOP trial does not settle the issue of anticoagulation for SCAF patients. Also, still unsettled by the LOOP trial (as well as by NOAH-AFNET 6 and ARTESIA) is whether the CHA_2_DS_2_-VASc score matters, whether the length of follow-up matters, and/or whether or how much the length of SCAF matters.

The NOAH-AFNET 6 trial^18^ was an investigator-initiated, event-driven, double-blind, double-dummy, randomized trial involving patients aged ≥65 years who had AHREs of ≥180 bpm lasting for ≥6 min on a PM, a defibrillator, or an ILR that had been implanted for ≥2 months and who had at least one additional risk factor for stroke but no known history of AF. Patients were randomly assigned in a 1:1 ratio to receive standard-dosing edoxaban or placebo (± 100 mg/day of aspirin if appropriate for any associated medical disorder, which was taken by about half of the patients in the placebo/aspirin group). The primary efficacy outcome was a composite of CV death, stroke, or systemic embolism, evaluated in a time-to-first event analysis. The safety outcome was a composite of death from any cause or major bleeding. The analysis population included 2536 patients, broken out to 1270 in the edoxaban group and 1266 in the placebo group. The mean age was 78 years, 37.4% of patients were women, the mean CHA_2_DS_2_-VASc score was 4 points, and the median duration of AHREs was 2.8 h. Approximately 10% of patients had a history of a prior stroke; no information was provided regarding prior major bleeding events.

After a median follow-up of 21 months and completion of the planned enrollment, the trial was terminated early due to both safety concerns and the results of an independent, informal assessment of futility for the efficacy of edoxaban. Of the 1270 patients on edoxaban, 232 (8.7%/year) developed clinically recognized AF, and 134 withdrew consent. Of the 1266 patients not given edoxaban, 230 developed clinically recognized AF (8.8%/year), and another 134 withdrew consent. A primary efficacy outcome event occurred in 83 patients (3.2%/patient-year) in the edoxaban group and 101 patients (4.0%/patient-year) in the placebo group (HR, 0.81; *P* = .15). The incidence of stroke approximated 1%/patient-year in both groups (0.9% vs. 1.1%). The results were not statistically different when examined by tertile of SCAF duration, by a CHA_2_DS_2_-VASc score of <5 points versus ≥5 points, or by use versus non-use of aspirin. A safety outcome event occurred in 149 patients (5.9%/patient-year) in the edoxaban group and 114 patients (4.5%/patient-year) in the placebo group (HR, 1.31; *P* = .03). Thus, among patients with AHREs detected by implantable devices in NOAH-AFNET 6, anticoagulation with edoxaban did not significantly reduce the incidence of a composite of CV death, stroke, or systemic embolism as compared to placebo, and the incidence of stroke was low in both groups. However, edoxaban did result in a greater incidence of a composite of death or major bleeding (deaths, 4.3% vs. 3.7%/patient-year; major bleeds, 2.1% vs. 1.0%/patient-year).^18^ Thus, while patients on edoxaban in NOAH-AFNET 6 had a numerically lower rate of stroke, systemic embolism, and CV mortality than those receiving the placebo (± aspirin), the difference was only 0.8%/patient-year, while, simultaneously, patients on edoxaban had numerically higher rates of total mortality (about 0.6%/patient-year) and major bleeding (about 1.1%/patient-year). Notably, the placebo limb of this trial was confounded by the use of aspirin in about half of the patients.

ARTESIA^19^ is the most recently reported of the three trials. ARTESIA was a prospective, multicenter, controlled trial of patients with SCAF detected by an implanted PM, a defibrillator, or an ILR who had additional risk factors for stroke and whose SCAF lasted anywhere from 6 min to a maximum of 24 h. A total of 4012 patients were enrolled. Patients were randomly assigned using a double-blind, double-dummy design to receive apixaban (n = 2015) at a dose of 5 mg twice daily (or 2.5 mg twice daily as indicated) or 81 mg of aspirin (n = 1997) daily. The trial medication was discontinued and anticoagulation was started if SCAF lasting >24 h or clinical AF developed. The mean age of patients was 77 years, the mean CHA_2_DS_2_-VASc score was 3.9 points, and 36% of patients were women. A history of a prior stroke, systemic embolism, or transient ischemic attack was reported by 9% of patients, while 2.5% had a prior history of major bleeding.

The primary efficacy outcome, stroke or systemic embolism, was assessed by an intention-to-treat (ITT) analysis (involving all the patients who had undergone randomization). The primary safety outcome, major bleeding, was assessed in the on-treatment population—that is, in all patients who had undergone randomization and received at least one dose of the assigned trial drug (with follow-up censored 5 days after permanent discontinuation of trial medication for any reason). After a mean follow-up of 3.5 ± 1.8 years, it was determined that stroke or systemic embolism had occurred in 55 patients in the apixaban group (0.78%/patient-year) and 86 patients in the aspirin group (1.24%/patient-year) (HR, 0.63; 95% confidence interval [CI], 0.45–0.88; *P* = .007). Total deaths were 4.3%/patient-year and 3.7%/patient-year for apixaban and aspirin, respectively. Thus, the rates of stroke or systemic embolism plus death appeared to be approximately 5% for each group, this being only minimally different from those seen in NOAH-AFNET 6. In the on-treatment population, the rate of major bleeding was 1.71%/patient-year in the apixaban group and 0.94%/patient-year in the aspirin group (HR, 1.80; 95% CI, 1.26–2.57; *P* = .001). Fatal bleeding occurred in five patients in the apixaban group and eight patients in the aspirin group. The rates of total mortality plus major bleeding (the safety endpoint used in NOAH-AFNET 6) appeared to be 6.4%/patient-year and 4.7%/patient-year, respectively, for apixaban and aspirin, which are again very similar to those seen in NOAH-AFNET 6. Thus, while patients on apixaban in ARTESIA had a statistically significant lower rate of stroke and systemic embolism than those on aspirin, as did patients on edoxaban versus placebo (± aspirin) in NOAH-AFNET 6, numerically, the difference was <0.5%/patient-year, while, simultaneously, patients on apixaban had numerically higher rates of total mortality (about 0.6%/patient-year) and major bleeding (about 0.8%/patient-year) (again, similar to the findings in NOAH-AFNET 6).

Taken together, LOOP, NOAH-AFNET 6, and ARTESIA were unable to provide a supportive picture of any real benefit for anticoagulation of patients with SCAF/AHREs. Thromboembolic event rates were low (stroke and systemic embolism rates were only about 1% in both NOAH-AFNET 6 and ARTESIA) and less than rates in the aspirin arms of major anticoagulation trials for clinically recognized AF. Moreover, reductions in thromboembolic event rates with the anticoagulation regimens studied were unimpressively small. Though some were statistically significant, they must be balanced against the off-setting risks of a slightly higher number of deaths and major bleeds in these same trials. Thus, the results of these trials question the existence of any risk-balanced value for preventative anticoagulation in SCAF patients, such as those enrolled. If further studies are performed in this space, at a minimum, they should be designed: (1) to assess how SCAF durations/AF burden may affect these results, by studying much larger populations to allow adequate stratification of duration and overall AF burden, as well as to determine whether longer AF runs/higher AF burden are just correlates of more severe atrial myopathy and how close the correlation is and (2) to assess whether using CHA_2_DS_2_-VASc scores numerically is an adequate means of assessing risk, as these scores are only based on the presence/absence of the abnormality contributing to the score and don’t consider the magnitude of effects it may have had in creating an embolic-prone atrial myopathy.^20^ Finally, these studies should remind us that trial results should be examined not only by considering changes in relative risk or HRs but also by appreciating the actual numerical differences between arms. If a study reduced an event rate from 2%/patient-year to 1%/patient-year, the relative risk reduction would be 50%, which might seem substantial, while the actual difference would be only 1%, which is a change that might seem quite minimal. In the aforementioned three trials, the small numerical differences need to be kept in mind when considering the data from them in discussions with patients and clinical decision-making.

Regardless of the above, based upon anticoagulation initiation in several ILR-detection trials for SCAF, such as Incidence of Previously Undiagnosed Atrial Fibrillation Using Insertable Cardiac Monitors in a High-risk Population (REVEAL AF) (56.3%),^21^ Predicting Determinants of Atrial Fibrillation or Flutter for Therapy Elucidation in Patients at Risk for Thromboembolic Events (PREDATE AF) (65.5%),^22^ Prevalence of Sub-clinical Atrial Fibrillation Using an Implantable Cardiac Monitor (ASSERT II) (26.2%),^23^ and others, all of which predate LOOP, NOAH-AFNET 6, and ARTESIA, many physicians appear to have already developed a belief that treatments for SCAF should mirror those for clinically recognized AF. One might wonder whether or not this belief played a role in the slower-than-expected enrollment rates in the abovementioned trials. Whether or not the results of these three trials will alter this behavior remains to be seen. Herein, recall the preamble to this paper. Clearly, additional investigation is required.

## Recent insights regarding anti-arrhythmic drugs versus catheter ablation for the rhythm management of atrial fibrillation

During the last two decades, a growing number of trials comparing CA to AAD therapy as a first-line treatment for AF or as a therapy following a failed first ablation have been performed.^4–6,8,24–53^ Most have enrolled patients with PAF or non-elderly patients without extremely large atria, although some have been done in older patients, patients with persistent AF, or patients with more advanced heart disease. Various methodological approaches for ablation have been used, and, likewise, the duration of pre-existing AF and other factors that can affect efficacy as well as approaches to post-ablation AF detection have varied. Nonetheless, in these trials, there has been growing consistency supporting CA as the superior approach for reducing recurrent AF. However, is the picture really that straightforward? I propose that it is not.

The primary efficacy outcome assessed in the debate of CA versus AADs with respect to rhythm-control efficacy for AF has most often been based on ITT analyses in the trials performed, although modified ITT and per-protocol and other analysis variations have also been used. An ITT analysis tests the outcome of one strategy of therapy versus another. In its purest sense, it does not matter whether a patient receives the assigned treatment, stays on it, or crosses over to the alternative treatment path when analyzed by ITT. However, clinicians treating patients do not practice by ITT. What the patients actually do during their treatment course—eg, stay on their treatment, discontinue their treatment, change to another therapy, add another therapy, alter a concomitant drug-interacting therapy—is vital to their outcome. Accordingly, it should be of great interest to recognize that in many/most of the recent CA versus AAD trials for AF rhythm control, not infrequently, patients in the CA treatment arm either continue or are started/restarted on an AAD post-ablation, though the outcomes assessed by ITT do not consider this hybridization. For those ablationists who consider AADs a relic of the past, consider here too the preamble to this paper. **Table 1** shows data from some recent trials that support this observation. Hence, it is important for clinicians who use the data from CA versus AAD trials in their practice to recognize that, very often, the apparent superiority of CA might be better categorized as CA plus an AAD rather than one versus the other.

## Additional details regarding the use of anti-arrhythmic drugs in patients who have catheter ablation for atrial fibrillation

While AADs and ablation both have important roles in the rhythm control of patients with AF, they should be recognized to have complementary rather than competing roles. Though incrementally effective over AADs for AF, ablation is far from perfect. AF recurrences post-ablation do occur. They may occur early, ie, during the early post-ablation “blanking period,” or they may occur thereafter.^4,45–53^ AADs have been used to suppress recurrences both during the “blanking period” as well as an alternative to repeat ablation for recurrences subsequent to the “blanking period.”

AAD suppression of AF during the “blanking period” does not predict freedom from recurrent AF thereafter if the AAD is discontinued. However, early recurrences have been associated with an increased incidence of late recurrences, and AAD continuation has reduced late recurrence rates. Recurrent AF, especially if symptomatic, can be treated by repeat ablative procedures and/or by the addition of AADs. **Table 1** suggests that the addition of AADs in this circumstance is relatively common. By way of example, one multicenter registry reported that maintaining AADs after the “blanking period” was associated with a long-term reduction in AF relapse—76% versus 86% at 1 year and 8%/year versus 14%/year thereafter.^47^ A study of 104 European centers reported that, for the 76% of patients who had successful ablation determined with 12 months of follow-up, 46% were still taking an AAD.^43^ Another multicenter, randomized, controlled study reported that patients taking an AAD post-ablation had a lower rate of repeat ablation (1.4% vs. 19.2%) and less unscheduled arrhythmia-related health care visits.^48^ In yet another report, among 682 consecutive patients with recurrent symptomatic recurrence after a first ablation for persistent AF, 40.1% of patients who had a second ablation and 46.2% of patients who did not continue to use AADs for rhythm control.^49^

Beyond these trials, we should also consider the very enlightening Pulmonary Vein Isolation with vs. Without Continued Anti-arrhythmic Drug Treatment in Subjects with Recurrent Atrial Fibrillation (POWDER AF) trial.^48^ POWDER AF was a randomized controlled trial that enrolled patients with PAF in whom previously ineffective AADs were continued during the 3-month “blanking period.” After the “blanking period” ended, patients who were free from AF were randomly assigned to either remain on their AAD or to discontinue it. The group that remained on AADs had significantly lower rates of recurrent atrial tachyarrhythmias (2.7% vs. 21.9%), repeat ablations (1.4% vs. 19.2%), and unscheduled arrhythmia-related health care visits (2.7% vs. 20.5%) during the 1-year follow-up period versus the group in whom AADs were stopped. Quality-of-life scores were similar in the two groups, and no serious adverse events occurred. Thus, POWDER AF clearly revealed a value to combining AADs with CA versus performing CA alone. How can such contributory efficacy-beneficial effects of AADs in post-CA patients not be considered when trials investigating CA versus AAD are analyzed or debated, given that, in many of them, AADs were used in CA patients and, similarly, some AAD patients crossed over to undergo CA?

Finally, in general, the same guideline-based algorithms for AAD selection pre-ablation should be held valid for their post-ablation selection. Importantly, AADs that were ineffective pre-ablation can be/have been effective post-ablation, where the post-ablation atria have been anatomically and electrophysiologically altered by the ablation. Notably, there have been no adequate prospective trials directly comparing AADs post-ablation, so their selection has been empiric—hopefully including a shared decision-making conversation with the patient. Perhaps with additional investigation, this information will also become available. If so, “stay tuned” for our 2024 update.

## Figures and Tables

**Table 1:** AAD Usage Post-catheter Ablation for AF

Study	Publication Year	Patients on AAD Post-ablation (%)*	Follow-up (months)
Kuck et al.^4^	2021	46.1%	36
Packer et al.^30^	2019	27%–45%	48
Aytemir et al.^32^	2013	21%	18
Packer et al.^33^	2013	26%	12
Willems et al.^34^	2019	29%–41%	12
Yu et al.^35^	2017	22% and 30%	12
Moltrasio et al.^36^	2021	18%	21
Park et al.^37^	2023	26%	48
Verma et al.^38^	2023	5%–10%	12
Kistler et al.^39^	2023	24%–29%	12
Zucchelli et al.^40^	2023	10%–30%	12
Yadav et al.^41^	2023	22%	10
Mortsell et al.^42^	2018	27%–39%	12
Arbello et al.^43^	2017	46%	12
Duytschaever et al.^44^	2018	23%–33%	12
Bertaglia et al.^45^	2017	42%–60%	12
Sohns et al.^53^	2023	29%	18

*Abbreviations:* AAD, anti-arrhythmic drug; AF, atrial fibrillation; f/u, follow-up. *After one versus multiple ablations. Depending on the specific ablation approach, depending upon the type of ablation set, and/or depending upon first-line ablation versus ablation in AAD-resistant patients.
